# Comparative efficacy of deep transcranial magnetic stimulation versus repetitive transcranial magnetic stimulation in improving lower extremity motor function in subacute stroke patients

**DOI:** 10.3389/fnagi.2025.1623039

**Published:** 2025-09-10

**Authors:** Chengshuo Wang, Linli Zhang, Mingyue Liu, Aomeng Xiang, Jingman Qi, Yanxin Fu, Ruoxuan Zhao, Zheyu Xiong, Liang Wu, Qin Zhang

**Affiliations:** ^1^Beijing Xiaotangshan Hospital, Beijing, China; ^2^School of Exercise and Health, Shanghai University of Sport, Shanghai, China; ^3^Tianjin Key Laboratory of Exercise Physiology and Sports Medicine, Institute of Sport, Exercise and Health, Tianjin University of Sport, Tianjin, China

**Keywords:** stroke, lower extremity, motor function, deep transcranial magnetic stimulation, repetitive transcranial magnetic stimulation

## Abstract

**Background:**

Deep transcranial magnetic stimulation (dTMS) is more beneficial in activating the leg muscle cortical representation. However, to date, no studies have evaluated the advantages of dTMS compared to repetitive transcranial magnetic stimulation (rTMS) in improving lower extremity motor function in subacute stroke patients. This study aims to compare the efficacy of dTMS and rTMS in treating lower extremity motor dysfunction in subacute stroke patients.

**Methods:**

In this single-blind, randomized controlled trial, fifty subacute stroke patients with lower extremity motor dysfunction were randomized to receive either dTMS or rTMS treatment. Patients’ Fugl-Meyer Assessment of Lower Extremity (FMA-LE), 10 m Maximum Walking Speed (10 m MWS), Berg Balance Scale (BBS), Timed Up and Go Test (TUGT), walking velocity, stride rate, stride length, gait cycle, double support percentage, and Resting Motor Threshold (RMT) were assessed before the intervention and after the 4-week intervention. Treatment effects were compared using two-way repeated-measures ANOVA. Correlations between lower extremity motor function and cortical excitability were analyzed using Pearson correlation analysis.

**Results:**

Forty-five patients completed the study (dTMS group: *n* = 22; rTMS group: *n* = 23). Two-way repeated measures ANOVA showed significant group × time interaction effects for FMA-LE, 10 m MWS, BBS, TUGT, walking velocity, stride length, gait cycle, and double support percentage. *Post hoc* analyses revealed both groups improved significantly from baseline in FMA-LE, 10 m MWS, BBS, TUGT, RMT, walking velocity, stride length, and double support percentage. The dTMS group additionally improved stride rate and gait cycle, while the rTMS group did not. Post-intervention, the dTMS group demonstrated significantly greater improvements than rTMS in FMA-LE, 10 m MWS, TUGT, and walking velocity. After 4 weeks, RMT was significantly negatively correlated with FMA-LE, 10 m MWS, BBS, and walking velocity. RMT was positively correlated with TUGT.

**Conclusion:**

Both dTMS and rTMS can improve lower extremity motor dysfunction in subacute stroke patients. Compared to rTMS, dTMS may provide more facilitative and accelerative effects to promote FMA-LE, TUGT, 10 m MWS, and walking velocity. Therefore, as an adjunct to conventional rehabilitation therapies, dTMS is a valuable therapeutic option in stroke rehabilitation programs.

## 1 Introduction

Stroke is an acute cerebrovascular disease characterized by focal neurological deficits caused by various obstructions (ischemic) or ruptures (hemorrhagic) (GBD 2021 Stroke Risk Factor Collaborators, 2024). At present, stroke has become the second leading cause of death and one of the main causes of disability worldwide (Feigin et al., 2022). With the development of medical technology, the mortality rate of stroke has decreased year by year, but 72% of survivors still have lower extremity dysfunction, which affects the walking function of patients (Ng and Hui-Chan, 2010). Nearly 30% of stroke patients cannot walk normally even in the recovery stage, which greatly affects their social interaction and, in severe cases, leads to lifelong disability (Frenkel-Toledo et al., 2021). Therefore, improving the lower extremity motor function and restoring the ability to walk independently as soon as possible are urgent problems that many stroke patients are eager to solve. However, both drug therapies (Szelenberger et al., 2020) and traditional rehabilitation therapies (e.g., neurodevelopmental therapy (Langhammer and Stanghelle, 2011), proprioceptive neuromuscular facilitation (Eng and Tang, 2007), and electromyography biofeedback (Woodford and Price, 2007)) seem to have little effect on improving lower extremity motor function in stroke patients.

Repetitive transcranial magnetic stimulation (rTMS) is a non-invasive brain stimulation technique widely used in clinical practice. At high frequencies (≥5 Hz), cortical excitability increases, whereas at low frequencies (≤1 Hz), a long-term depression effect is produced, and cortical excitability decreases (Kim et al., 2020). Currently, rTMS has become an important adjuvant therapy in the rehabilitation of stroke patients, and its efficacy in improving upper extremity movement disorders (Li et al., 2024), cognitive impairment (Zhang et al., 2024), depression (Cappon et al., 2022), and other diseases (Lefaucheur et al., 2020) has been confirmed. Its application in the rehabilitation of lower extremity function after stroke has also achieved initial results (Tung et al., 2019; Fan et al., 2021). However, the therapeutic effect of traditional rTMS for lower extremity motor function after stroke may have a certain upper limit because the primary motor cortex (M1) leg area is located deep within the intercerebral fissure 3–4 cm from the scalp surface, which makes it challenging for the circular coil or figure-of-eight coil of rTMS to provide magnetic stimulation to the M1 leg functional area to intervene (Kakuda et al., 2013). In contrast, deep transcranial magnetic stimulation (dTMS) using the H-coil can effectively overcome this depth-related stimulation challenge.

Deep transcranial magnetic stimulation is an emerging non-invasive brain stimulation technique developed on the basis of rTMS. Currently, dTMS is used to study and treat various mental and neurological diseases (Roth et al., 2014a). Compared with the traditional figure-of-eight coil, the H-coil used in dTMS can stimulate deeper areas of the brain without increasing the stimulation intensity (Ferrulli et al., 2021), including deeper cortical regions and fibers targeting subcortical regions (Zangen et al., 2005; Roth et al., 2014b) and allows the stimulation of the cortical representation of distal lower extremity muscles to be possible at lower intensities than the figure-of-eight coil (Roth et al., 2002, 2014b). The electric field generated by dTMS provides the possibility to stimulate the lower extremity representation in the M1 (Chieffo et al., 2016). At present, studies have explored the comparison of the efficacy of dTMS and sham stimulation in improving lower extremity motor dysfunction in stroke patients (Chieffo et al., 2014, 2021). Both studies have found that high-frequency dTMS lasting for 3 weeks can significantly improve lower extremity motor function in stroke patients compared with sham stimulation. However, although dTMS is superior to sham stimulation, it is unclear whether this technique is superior to traditional rTMS. Therefore, the main purpose of this study is to compare the efficacy of dTMS and rTMS in treating lower extremity motor dysfunction in subacute stroke patients and to provide a scientific and reasonable basis for the treatment of lower extremity motor dysfunction in such patients.

## 2 Materials and methods

### 2.1 Study design

In this single-blind, randomized controlled trial, participants were randomly assigned to either the dTMS or the rTMS group. Before the intervention, we collected patients’ demographic characteristics (including age, gender, course of the disease, stroke type, lesion side, etc.) and conducted baseline assessments of lower extremity motor function (including lower extremity motor ability, balance function, gait parameters, etc.). A 4-week intervention was subsequently administered. After the completion of all interventions, participants underwent reassessment of lower extremity motor function. The study was conducted at Beijing Xiaotangshan Hospital between January and November 2024. The trial protocol was approved by the Ethics Committee of Beijing Xiaotangshan Hospital (No. 2024-01) and was registered at the Chinese Clinical Trial Registry (Trial registration number: ChiCTR2400081419). All subjects signed a written informed consent form before initiating the trial.

### 2.2 Sample size calculation

FMA-LE was used as the primary outcome measure. According to the results of a previous study (Mo and Liu, 2020), it was assumed that the mean values of FMA-LE in the dTMS group and the rTMS group were 27.15 and 24.69, respectively, and the standard deviation was 2.64. The significance level (α) was set at 0.05, and the statistical power was set at 0.80. The sample size N1 = 20 in the dTMS group and N2 = 20 in the rTMS group were calculated by PASS 15 software (NCSS Corp, Kaysville, UT, USA). The final sample size required was 25 per group to allow for a 20% dropout rate. A total of at least 50 patients were included.

### 2.3 Setting, recruitment and participants

We recruited a total of 50 subacute stroke patients. The patient inclusion criteria were as follows: (1) patients who were diagnosed with cerebral hemorrhage or cerebral infarction by head CT and/or MRI, with motor dysfunction of lower limbs; (2) ischemic or hemorrhagic stroke for the first time; (3) >2 weeks and <6 months after stroke onset; (4) aged between 30 and 75 years; (5) patients with standing balance ≥1 level; (6) patients who were able to complete 10 m walking with assistance; (7) patients who voluntarily completed dTMS or rTMS treatment and signed informed consent. Exclusion criteria included: (1) patients with a metallic foreign body in the skull, a cardiac pacemaker, or a cochlear implant; (2) patients with a history of epilepsy; (3) patients with severe heart, lung, liver, kidney and other vital organ failure; (4) patients with severe cognitive, communication, or emotional disorders; (5) patients who had received dTMS or rTMS treatment within the first 3 months of this study.

### 2.4 Interventions

Both groups of patients received routine treatment, including using drugs to inhibit platelet aggregation, lipid regulation, blood pressure control, blood glucose control, etc. At the same time, they all participated in regular physical therapy for individual lower extremity motor function (5 days/week for a total of 4 weeks). This training includes transfer, sitting, standing, static and dynamic balance, and walking training.

**FIGURE 1:**
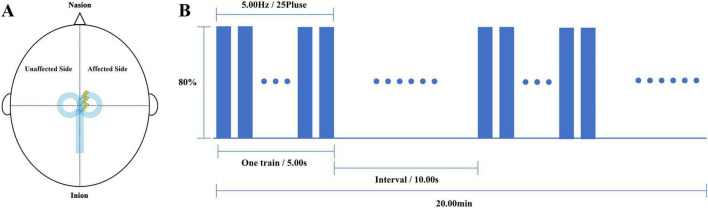
Stimulation methods: **(A)** Stimulation target diagram, **(B)** stimulation paradigm.

Deep transcranial magnetic stimulation or Repetitive transcranial magnetic stimulation therapy was completed before each physical therapy in both groups. We used a Brainsway dTMS system equipped with an H7-coil (Brainsway Ltd, Jerusalem, Israel) to intervene in patients in the dTMS group. The optimal stimulation site on the skull was defined as the position on the midsagittal plane at which the largest motor evoked potential (MEP) in the tibialis anterior (TA) of the unaffected lower extremity was elicited on surface electromyography. The coil was positioned with its center vertically over the determined stimulation site on the midsagittal plane, so that the bilateral leg motor areas would be stimulated simultaneously (Figure 1A). Stimulation parameters were: 80%–120% of RMT (increasing from 80%); 80 5-s trains at 5 Hz, 10-s inter-train interval, with a total of 2000 pulses over 20 min (Figure 1B). The stimulation was conducted once a day for 5 days per week for 4 weeks. We used the M-100 Ultimate Transcranial Magnetic Stimulation device equipped with a 70-mm figure-of-eight coil from Shenzhen Yingzhi Technology Co., Ltd. (China) to intervene in patients in the rTMS group. The stimulation target and treatment parameters were the same as those in the dTMS group.

### 2.5 Outcome measures

The demographic data were obtained from the medical files. A blinded therapist, who was not involved in the participant selection process, administered the Fugl-Meyer Assessment of Lower Extremity (FMA-LE), 10 m Maximum Walking Speed (10 m MWS), Berg Balance Scale (BBS), Timed Up and Go Test (TUGT), walking velocity, stride rate, stride length, gait cycle, double support percentage, Motor Evoked Potential (MEP), and Resting Motor Threshold (RMT) before and after the 4-week intervention.

The FMA-LE includes 7 major items, such as reflex, hip movement, knee movement, and ankle movement, with a total of 17 items and a total score of 34 points. The higher the score, the better the recovery of lower extremity motor function of patients. The content of the scale is detailed, which can accurately reflect the recovery of lower extremity motor function in stroke patients with hemiplegia (Hsieh et al., 2009).

Assessment of 10 m MWS: The starting point, 2 m, 8 m, and the endpoint were marked on the ground with a straight distance of 10 m. After hearing the beginning command, the patient moved from the starting point to the endpoint at the fastest speed. The evaluator used a stopwatch to record the time required for the patient to step from 2 m to 8 m and calculated the 10 m MWS. The test was carried out three times, and the average value of the data obtained three times was recorded. The faster the patient’s walking speed, the better the patient’s walking function.

The BBS is the most widely used clinical scale to assess balance performance in patients with neurological disorders, including static balance and dynamic balance. There are 14 items in the BBS. The lowest score of each item is 0, the highest score is 4, and the total score is 56. Higher scores indicate better balance function (Meseguer-Henarejos et al., 2019).

The TUGT is a rapid quantitative assessment method for body mobility ability, balance function, and fall risk. Procedure: The subjects sat in a chair with armrests and backrests, and the evaluators recorded the time (in seconds) that the subjects left the back of the seat and walked forward for 3 m, then turned around to sit down and leaned back against the chair back. The test was carried out three times, and the average value of the data obtained three times was recorded. The shorter the time, the better the balance function (Flansbjer et al., 2005).

Gait parameters were assessed by the whole body three-dimensional gait and motion analysis system (Jiangsu Neucognic Medical Co., Ltd). The patient wore the measuring device and walked 10 m until the assessment steps were fully mastered before starting the formal test. The walking velocity, stride rate, stride length, gait cycle, and double support percentage of the two groups before and after intervention were measured.

Assessment of RMT: We used single-pulse TMS with a double-cone coil to stimulate the M1 leg area and gradually decreased the stimulation intensity until RMT was confirmed, defined as eliciting an MEP of at least 50 μV amplitude in the relaxed tibialis anterior muscle of the unaffected side in a minimum of 5 out of 10 trials (Lefaucheur et al., 2020). Assessment of MEP: First, the MEP status on the affected side was determined. The double-cone coil was placed over the M1 leg area, and suprathreshold stimulation at 120% RMT intensity was delivered. MEPs were recorded from the tibialis anterior muscle on the affected side. If MEPs with normal amplitude and consistent latency were observed in at least 10 single-pulse TMS stimuli, the result was considered MEP+. Otherwise, it was MEP−. After confirming MEP + status, MEPs elicited by 10 single-pulse TMS stimuli were recorded, and their average latency and amplitude were calculated (Burke et al., 2019).

### 2.6 Blinding and randomization

Computer-generated random sequences were used, and the random numbers were hidden in opaque numbered envelopes and opened in numerical order by an uninvolved researcher. Fifty participants were randomly allocated to 2 groups in a 1:1 ratio and received either dTMS or rTMS. Recruitment personnel, data collectors, and statistical analysts were blinded to the group allocation, with a designated researcher being responsible for intervention based on the group assignments.

### 2.7 Statistical analysis

The normality of distribution was assessed using the Shapiro-Wilk normality test. Measurement data that follow a normal distribution are expressed as mean ± standard deviation (SD). Count data are presented as numbers (*n*) and percentages (%). Pearson’s chi-squared test or Fisher’s exact test was used to compare the count data. The independent samples *t*-test was applied to compare the measurement data of the subjects in the two groups before the intervention. When the data met the assumptions of normality and homogeneity of variances, two-way repeated measures ANOVA was used to investigate the effects of group (dTMS vs. rTMS) and time (pre-test vs. post-test) on lower extremity motor ability, balance function, gait parameters, and cerebral cortical excitability. If there was an interaction, a simple effects *post hoc* analysis was further carried out. Statistical analyses were performed using SPSS (version 26.0; IBM, Armonk, NY, USA). The significance level (α) was set at 0.05, and the effect size was represented by η^2^. The Pearson correlation analysis was conducted to identify whether there were correlations between lower extremity motor function and cerebral cortical excitability at 4 weeks after interventions.

## 3 Results

### 3.1 Study participation

**FIGURE 2:**
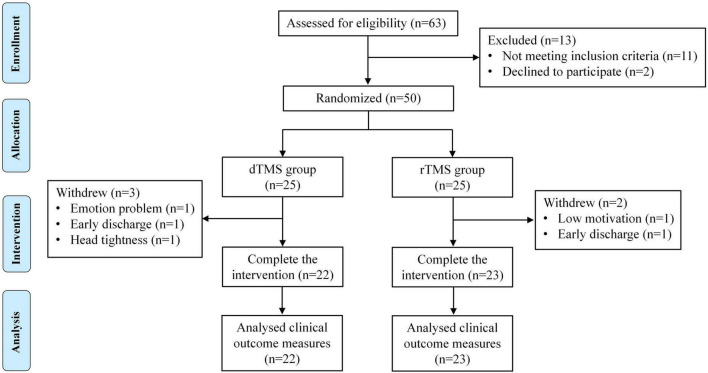
Flow diagram of the randomized controlled trial.

From January to November 2024, a total of 63 patients were screened for participation in this study. Among them, 11 people did not meet the inclusion criteria, and the other 2 people refused to participate in the study for personal reasons. Finally, a total of 50 patients were included in this study and randomly assigned to the dTMS group (*n* = 25) and the rTMS group (*n* = 25) in a 1:1 ratio. Three patients in the dTMS group withdrew: one patient withdrew from the study due to emotion problem, one patient decided to be discharged from the hospital for personal reasons, and one patient withdrew from the study due to head tightness caused by a large head circumference. Two patients in the rTMS group withdrew: one patient withdrew from the study due to low motivation, and the other patient decided to be discharged from the hospital for personal reasons. The remaining patients (*n* = 45) completed the study as expected (Figure 2).

Finally, the dTMS group included 18 males and 4 females, with an average age of 60.32 ± 11.30 years. The rTMS group included 17 males and 6 females, with an average age of 61.91 ± 10.63 years. No significant differences in demographic characteristics were found between the groups (*p* > 0.05) (Table 1). During the study, there were no serious adverse events in all patients, with one patient reporting mild nausea in the dTMS group and one reporting mild headache in the rTMS group. After a short day of rest, the discomfort in both patients was relieved. No statistically significant difference in side effects was found between the two groups (*p* > 0.05).

**TABLE 1:** Demographic characteristics at baseline.

Stimulation methods	dTMS group (*n* = 22)	rTMS group (*n* = 23)	Effect size	*p-*value
Age, (years)	60.32 ± 11.30	61.91 ± 10.63	−0.488	0.628
Gender, *n* (%)				0.780
Male	18 (81.82)	17 (73.91)		
Female	4 (18.18)	6 (26.09)		
Course of the disease, (days)	56.05 ± 24.52	51.22 ± 23.09	0.680	0.453
Stroke type, *n* (%)			0	1.000
Ischemic	19 (86.36)	20 (86.96)		
Haemorrhagic	3 (13.64)	3 (13.04)		
Lesion side, *n* (%)			0.218	0.641
Left	13 (59.09)	12 (52.17)		
Right	9 (49.91)	11 (47.83)		
HR (bpm)	75.68 ± 9.76	80.57 ± 7.61	−1.877	0.067
SBP (mmHg)	134.86 ± 13.79	135.48 ± 16.44	−0.136	0.893
DBP (mmHg)	78.73 ± 10.99	77.57 ± 9.82	0.374	0.710
Drinking, *n* (%)	15 (68.18)	14 (60.87)	0.262	0.608
Smoking, *n* (%)	8 (36.36)	4 (17.39)	2.070	0.150
**Comorbidities**				
Hypertension, *n* (%)	19 (86.36)	20 (86.96)	0	1.000
Hyperlipemia, *n* (%)	13 (59.09)	17 (73.91)	1.112	0.292
Diabetes, *n* (%)	8 (36.36)	15 (65.22)	3.611	0.057

dTMS, deep transcranial magnetic stimulation; rTMS, repetitive transcranial magnetic stimulation; HR, heart rate; SBP, systolic blood pressure; DBP, diastolic blood pressure.

### 3.2 Lower extremity motor ability

**FIGURE 3:**
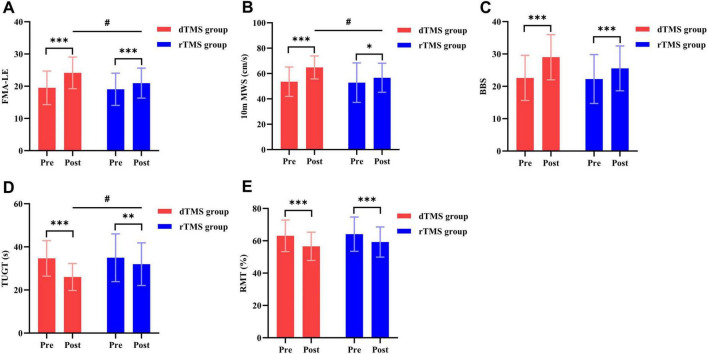
Effects of dTMS and rTMS on lower extremity motor ability, balance function, and cerebral cortical excitability. **(A)** Fugl-Meyer Assessment of Lower Extremity (FMA-LE); **(B)** 10-meter Maximum Walking Speed (10 m MWS); **(C)** Berg Balance Scale (BBS); **(D)** Timed Up and Go Test (TUGT); **(E)** Resting Motor Threshold (RMT). # indicates significant differences between groups after 4 weeks of intervention. * indicates significant differences within groups (*p* < 0.05). ** indicates significant differences within groups (*p* < 0.01). *** indicates significant differences within groups (*p* < 0.001).

The two-way repeated measures ANOVA revealed a significant interaction effect between group and time for FMA-LE (*F* = 35.534, *p* < 0.001, η^2^ = 0.452, Table 2) and 10 m MWS (*F* = 16.156, *p* < 0.001, η^2^ = 0.273, Table 2). *Post hoc* analyses demonstrated that compared to baseline, the dTMS group showed significant improvements in FMA-LE (*p* < 0.001, Figure 3A) and 10 m MWS (*p* < 0.001, Figure 3B) post-intervention, while the rTMS group also exhibited significant enhancements in FMA-LE (*p* < 0.001, Figure 3A) and 10 m MWS (*p* = 0.010, Figure 3B). After 4 weeks of intervention, significant between-group differences were observed in FMA-LE (*p* = 0.031, Figure 3A) and 10 m MWS (*p* = 0.012, Figure 3B), favoring the dTMS group.

**TABLE 2:** Changes in clinical outcome measures.

Variables	dTMS group (*n* = 22)		rTMS group (*n* = 23)		Time × group		
	Pre	Post	Pre	Post	*F*	*p*	η^2^
**Lower extremity motor ability**							
FMA-LE	19.50 ± 5.23	24.14 ± 4.92*^#^	19.04 ± 5.00	20.96 ± 4.66*	35.534	<0.001	0.452
10 m MWS, cm/s	53.55 ± 11.56	64.80 ± 9.10*^#^	52.80 ± 15.60	56.66 ± 11.47*	16.156	<0.001	0.273
**Balance function**							
BBS	22.59 ± 6.98	29.00 ± 6.99*	22.26 ± 7.55	25.56 ± 6.93*	26.757	<0.001	0.384
TUGT, s	34.66 ± 8.24	26.04 ± 6.26*^#^	34.96 ± 11.07	31.99 ± 9.91*	22.756	<0.001	0.346
**Gait parameters**							
Walking velocity, cm/s	39.32 ± 9.86	49.81 ± 7.85*^#^	38.69 ± 12.87	42.30 ± 12.07*	34.830	<0.001	0.448
Stride rate, steps/min	68.32 ± 13.74	76.83 ± 14.92*	68.08 ± 16.24	72.17 ± 19.70	3.182	0.082	0.069
Stride length, cm	65.97 ± 14.41	75.14 ± 13.63*	65.59 ± 18.18	69.63 ± 17.80*	7.525	0.009	0.149
Gait cycle, s	1.83 ± 0.37	1.62 ± 0.32*	1.88 ± 0.52	1.80 ± 0.55	5.349	0.026	0.111
Double support percentage,%	38.40 ± 11.77	32.39 ± 9.00*	39.26 ± 13.03	36.27 ± 11.06*	6.010	0.018	0.123
**Cortical excitability**							
RMT,%	63.09 ± 9.77	56.55 ± 8.74*	64.09 ± 10.59	59.22 ± 9.34*	3.280	0.077	0.071

FMA-LE, Fugl-Meyer Assessment of Lower Extremity; 10 m MWS, 10-meter Maximum Walking Speed; BBS, Berg Balance Scale; TUGT, Timed Up and Go Test; RMT, Resting Motor Threshold; MEP, motor evoked potential. ^#^Indicates significant differences between groups after 4 weeks of intervention. *Indicates significant differences within groups (*p* < 0.05).

### 3.3 Balance function

The two-way repeated measures ANOVA revealed a significant interaction effect between group and time for BBS (*F* = 26.757, *p* < 0.001, η^2^ = 0.384, Table 2) and TUGT (*F* = 22.756, *p* < 0.001, η^2^ = 0.346, Table 2). *Post hoc* analyses demonstrated that compared to baseline, the dTMS group showed significant improvements in BBS (*p* < 0.001, Figure 3C) and TUGT (*p* < 0.001, Figure 3D) post-intervention, while the rTMS group also exhibited significant enhancements in BBS (*p* < 0.001, Figure 3C) and TUGT (*p* = 0.001, Figure 3D). After 4 weeks of intervention, a significant between-group difference was observed in TUGT (*p* = 0.021, Figure 3D), favoring the dTMS group. However, no significant between-group differences were detected in BBS (*p* > 0.05, Figure 3C).

### 3.4 Gait parameters

**FIGURE 4:**
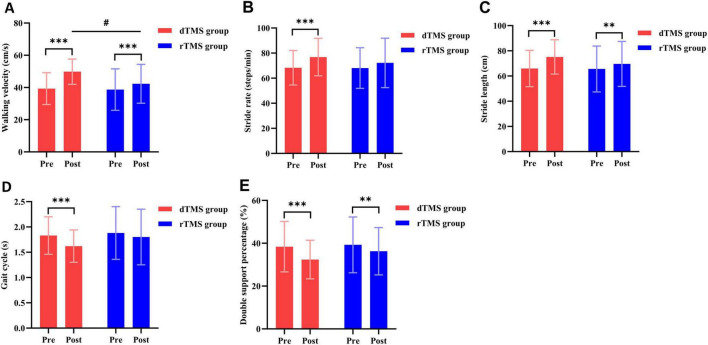
Effects of dTMS and rTMS on gait parameters. **(A)** Walking velocity; **(B)** stride rate; **(C)** stride length; **(D)** gait cycle; **(E)**, double support percentage. # indicates significant differences between groups after 4 weeks of intervention. **Indicates significant differences within groups (*p* < 0.01). *** indicates significant differences within groups (*p* < 0.001).

The two-way repeated measures ANOVA revealed no significant interaction effect between group and time for stride rate (*p* > 0.05, Table 2). However, significant interactions were observed for walking velocity (*F* = 34.830, *p* < 0.001, η^2^ = 0.448, Table 2), stride length (*F* = 7.525, *p* = 0.009, η^2^ = 0.149, Table 2), gait cycle (*F* = 5.349, *p* = 0.026, η^2^ = 0.111, Table 2), and double support percentage (*F* = 6.010, *p* = 0.018, η^2^ = 0.123, Table 2). *Post hoc* analyses indicated that compared to baseline, the dTMS group showed significant improvements in walking velocity (*p* < 0.001, Figure 4A), stride rate (*p* < 0.001, Figure 4B), stride length (*p* < 0.001, Figure 4C), gait cycle (*p* < 0.001, Figure 4D), and double support percentage (*p* < 0.001, Figure 4E) post-intervention. The rTMS group also showed improvements in walking velocity (*p* < 0.001, Figure 4A), stride length (*p* = 0.003, Figure 4C), and double support percentage (*p* = 0.002, Figure 4E). After 4 weeks of intervention, a significant between-group difference was found only in walking velocity (*p* = 0.018, Figure 4A), favoring the dTMS group.

### 3.5 Nervous system function

The two-way repeated measures ANOVA revealed no significant interaction effect between group and time for resting motor threshold (RMT) (*p* > 0.05, Table 2), but a significant main effect of time was observed (*p* < 0.001). Within-group analyses revealed significant post-intervention improvements in RMT for both the dTMS and rTMS groups compared to baseline (*p* < 0.001, Figure 3E). Prior to intervention, MEPs (hemiplegic side) were elicitable in 4 subjects in the dTMS group and 3 subjects in the rTMS group, with no between-group difference in MEP elicitation rates *(p* > 0.05). After 4 weeks of intervention, MEPs were elicitable in 8 subjects (18.18% increase) in the dTMS group and 5 subjects (8.70% increase) in the rTMS group. However, no significant between-group difference in post-intervention MEP elicitation rates was observed (*p* > 0.05).

### 3.6 Correlation analysis between the lower extremity motor function and the cerebral cortical excitability

**FIGURE 5:**
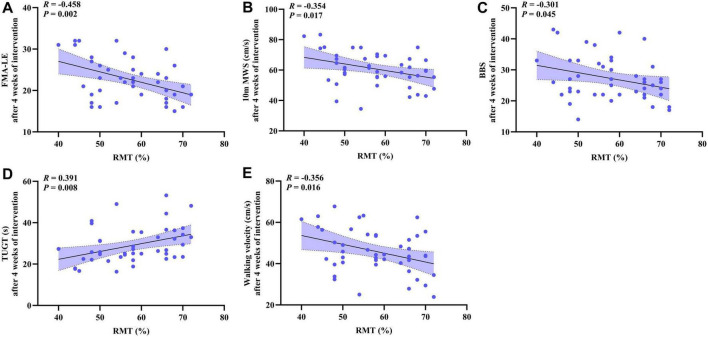
The scatter plot shows the correlation analysis between the lower extremity motor function (FMA-LE, 10 m MWS, BBS, TUGT, walking velocity) and the cerebral cortical excitability (RMT) after 4 weeks of intervention. **(A)** FMA-LE and RMT; **(B)** 10 m MWS and RMT; **(C)** BBS and RMT; **(D)** TUGT and RMT; **(E)** Walking velocity and RMT.

The correlation between lower extremity motor function and motor cortex excitability at 4 weeks post-intervention was explored. Statistical analysis showed that RMT was significantly negatively correlated with FMA-LE (*R* = −0.458, *p* = 0.002, Figure 5A), 10 m MWS (*R* = −0.354, *p* = 0.017, Figure 5B), BBS (*R* = −0.301, *p* = 0.045, Figure 5C), and walking velocity (*R* = −0.356, *p* = 0.016, Figure 5E). RMT was positively correlated with TUGT (*R* = 0.391, *p* = 0.008, Figure 5D).

## 4 Discussion

This is the first randomized controlled trial comparing the efficacy of dTMS and rTMS in treating lower extremity motor dysfunction in subacute stroke patients. The results showed that both dTMS and rTMS improved lower extremity motor ability, balance function, gait parameters, and cerebral cortical excitability in subacute stroke patients compared to baseline. However, dTMS provided more facilitative and accelerative effects than rTMS in improving FMA-LE, TUGT, 10 m MWS, and walking velocity. Consistent with previous findings (Chieffo et al., 2014, 2021), no serious side effects were observed during dTMS intervention, indicating its safety and effectiveness.

Under normal circumstances, the two cerebral hemispheres regulate each other’s excitability through connections via the corpus callosum, thereby maintaining a balance between them (Duque et al., 2005). However, in stroke patients, there is an imbalance in interhemispheric inhibition (Xu et al., 2019), which leads to impaired excitability in the motor cortex of the affected hemisphere and impacts limb movement (Liepert et al., 2000). The potential mechanism of rTMS improving motor function in stroke patients is based on the interhemispheric competition (IHC) model (Nowak et al., 2009). However, it is important to note that the applicability of the IHC model to post-stroke lower limb functional recovery has recently been questioned. This is due to significant differences between post-stroke lower and upper limb hemiparesis that involve the control of nerve fibers. Thus, directly extrapolating the IHC model from upper limb rehabilitation to lower limb rehabilitation may not be justified. Studies have found that, in healthy individuals, approximately 90% or more of upper extremity motor function is innervated by neural fibers from the contralateral hemisphere. However, 70%–80% of lower extremity motor function is governed by neural fibers from the contralateral hemisphere, while the remaining 20%–30% is controlled by nerve fibers from the ipsilateral hemisphere (Luft et al., 2002). Therefore, the lower extremity representation in the M1 of the contralesional hemisphere contributes to motor functional recovery of the affected lower extremity after stroke. Additionally, Enzinger et al. using functional magnetic resonance imaging (fMRI), observed that improvements in walking function are associated with increased brain activation in bilateral M1, the cingulate motor area, the caudate nucleus, and the thalamus on the affected side. Therefore, these studies collectively suggest that the “bilateral facilitation model” for the lower extremity representation in the M1 seems more scientific. At the same time, excitatory stimulation targeting the lower extremity representation in bilateral M1 has demonstrated potential efficacy in enhancing gait (Kakuda et al., 2013; Chieffo et al., 2014, 2021). Accordingly, the M1 leg area stimulation protocol employed in this study was based on the protocol described by Chieffo et al. (2021).

Several studies have individually demonstrated the efficacy of both dTMS and rTMS in improving lower extremity motor function compared to sham stimulation (Chieffo et al., 2014, 2021; Tung et al., 2019; Fan et al., 2021). In this study, we observed significant improvements in FMA-LE and 10 m MWS in both groups compared to baseline, with dTMS demonstrating greater efficacy than rTMS. We hypothesize that the primary reason for this phenomenon lies in the anatomical location of the lower extremity representation within the M1, which resides deep within the interhemispheric fissure (approximately 3–4 cm below the scalp surface) and is surrounded by the corpus callosum, cingulate gyrus, and falx cerebri. Traditional figure-of-eight coils can only stimulate the superficial cortex of the brain (typically reaching only 2.0–2.5 cm below the scalp surface). In contrast, under identical stimulation targets and intensities, H-coils can activate deeper motor cortical regions and influence broader neuronal pathways (Levkovitz et al., 2015). Furthermore, the dTMS device consists of a flexible base that matches the shape of the head and a coil element that is tangent to the scalp, which can minimize the accumulation of electrostatic charges on the brain surface and enhance the penetration of the coil into the deep brain (Tofts and Branston, 1991; Eaton, 1992). Roth et al. (2014b) compared the H-coil with the figure-of-eight coil and found that the H-coil demonstrated superior efficacy in activating cortical representations of leg muscles. These findings may support our results, suggesting that dTMS offers greater advantages over rTMS in enhancing lower extremity motor function.

Compared with healthy people, stroke patients have decreased walking velocity, stride rate, and stride length, as well as an imbalance between lower limbs (Hsu et al., 2003), resulting in abnormal gait that reduces walking and balance ability and increases the risk of falling (Wang et al., 2024). This study found that after 4 weeks of intervention, intergroup analysis revealed that dTMS was significantly superior to rTMS in enhancing walking velocity in subacute stroke patients (*p* < 0.05). The observed differences may be attributed not only to dTMS’s advantages in activating the lower extremity representation of the M1 and modulating deep neural circuits, as previously explained but also to the relatively longer central conduction pathways from the cerebral cortex to the lower limbs. This increased anatomical length raises the likelihood of temporal dispersion in corticospinal impulse waves. Consequently, higher-intensity cortical stimulation is required to synchronize motor neuron discharges innervating leg muscles, resulting in a higher activation threshold for leg muscles compared to hand muscles (Groppa et al., 2012). To achieve this goal using traditional figure-of-eight coils, increased stimulation intensity would be necessary. However, according to standard TMS safety guidelines, such high-intensity stimulation is neither safe nor permissible due to the risk of significant adverse effects. In contrast, dTMS ensures patient safety while delivering optimal stimulation efficacy.

Balance function is closely associated with post-stroke walking ability, functional independence, and fall risk. Therefore, restoring balance function as early as possible is one of the important goals of rehabilitation for stroke patients (Louie and Eng, 2018). Human balance is regulated by the brain through the integration of multisensory information. As a part of the frontal cortex-basal ganglia network, the M1 of the cerebral cortex is considered to be related to balance and posture control (Demain et al., 2014). Related studies have shown that TMS targeting the M1 not only modulates cortical excitability but also enhances neural network connections between the M1 and the cerebellum, supplementary motor area (SMA), and related functional areas (Tremblay et al., 2016). The enhancement of the connections between these different brain regions is of great significance in improving the balance ability and posture control ability of stroke patients. Our study found that after 4 weeks of intervention, dTMS was significantly superior to rTMS in improving the TUGT in subacute stroke patients (*p* < 0.05). Interestingly, no significant advantage of dTMS over rTMS was observed in BBS improvements. We hypothesize that this discrepancy may arise because dTMS exhibits greater efficacy in enhancing dynamic balance (e.g., rising, walking, turning), whereas the BBS primarily assesses global balance capacity and is less sensitive to subtle changes in specific dynamic functions (e.g., turning speed).

Improving cerebral cortical excitability is of great significance for reconstructing brain networks and facilitating descending cortical pathways (Bolognini et al., 2009). In this study, RMT was measured to reflect the excitability of the motor cortex. After 4 weeks of intervention, we found that both dTMS and rTMS could improve the excitability of the cerebral cortex in subacute stroke patients. MEP can reflect the conduction function and integrity of the corticospinal tract (Welch et al., 2020). In this study, because few patients exhibited elicitable MEPs on the hemiplegic side before intervention, we used the MEP elicitation rate to reflect the recovery of the corticospinal tract. The results showed no significant difference in the MEP elicitation rate between the two groups before and after intervention. This may be because the reconstruction of neural pathways may be affected by many factors, such as growth factors and inflammatory factors in the microenvironment and energy parameters and frequency parameters of external electromagnetic stimulation (Zheng and Xu, 2020). Furthermore, the single-target stimulation protocol used in this study may limit the activation of latent or impaired neural pathways. In the future, multi-target stimulation of neural pathways can be considered to further activate specific cortical areas or corticospinal tracts and regulate motor neural pathways related to reconstruction. Finally, the intervention period of this study is relatively short, and it can be extended in the future to explore the effect of dTMS on MEP and its potential mechanism.

Additionally, we found significant correlations between patients’ RMT and multi-dimensional assessments of lower extremity motor function (including FMA-LE, 10 m MWS, BBS, TUGT, and walking velocity) after 4 weeks of intervention. This suggests that cerebral cortical excitability may act as a critical mediating factor in the recovery of lower extremity motor function. Therefore, greater attention should be paid to changes in cerebral cortical excitability during clinical rehabilitation for stroke patients. Comparison with prior studies: Rosso and Lamy (2018) reported an association between RMT and upper limb motor function but did not involve lower limbs. Our study extends the predictive value of RMT to lower limb motor scenarios. Furthermore, RMT serves only as an indirect indicator of cerebral cortical excitability. Future research should integrate multimodal neuroimaging techniques, such as Transcranial Magnetic Stimulation-Electroencephalography (TMS-EEG) or Functional Magnetic Resonance Imaging (fMRI), to validate the relationship between brain network-level changes and lower extremity motor function. It should be noted that the results of this correlation study are exploratory findings, and their significance still needs to be interpreted cautiously in conjunction with specific clinical contexts.

This study had several limitations. First, most of the patients in this study were male, aged between 50 and 70 years old, which may lead to gender and age bias. Second, this study used neuroelectrophysiological techniques to observe the excitability of the cerebral cortex and the recovery of the central nervous system. However, there was still a lack of functional imaging techniques to verify. Third, we did not classify ischemic and hemorrhagic stroke, so it is unclear whether there are any differences in the efficacy of dTMS in patients with different stroke subtypes. Fourth, this study was evaluated only after the end of the intervention. In future studies, long-term follow-up evaluation should be added to clarify the persistence and stability of the intervention effect. Finally, since prior studies have confirmed a statistically significant difference in therapeutic efficacy between dTMS and sham stimulation, this study did not include a sham stimulation group. Therefore, it is impossible to directly compare the efficacy differences between dTMS and sham stimulation, and between rTMS and sham stimulation.

## 5 Conclusion

Both dTMS and rTMS can improve lower extremity motor dysfunction in subacute stroke patients. Compared to rTMS, dTMS may provide more facilitative and accelerative effects to promote FMA-LE, TUGT, 10 m MWS, and walking velocity. Therefore, as an adjunct to conventional rehabilitation therapies, dTMS is a valuable therapeutic option in stroke rehabilitation programs.

## Data Availability

The raw data supporting the conclusions of this article will be made available by the authors, without undue reservation.
